# Structural and Functional Analysis of Calcium Ion Mediated Binding of 5-Lipoxygenase to Nanodiscs

**DOI:** 10.1371/journal.pone.0152116

**Published:** 2016-03-24

**Authors:** Ramakrishnan B. Kumar, Lin Zhu, Helena Idborg, Olof Rådmark, Per-Johan Jakobsson, Agnes Rinaldo-Matthis, Hans Hebert, Caroline Jegerschöld

**Affiliations:** 1 Department of Biosciences and Nutrition, Karolinska Institutet, 14183 Huddinge, Sweden; 2 School of Technology and Health, KTH Royal Institute of Technology, 14183 Huddinge, Sweden; 3 Rheumatology Unit, Department of Medicine, Karolinska Institutet, 17176 Stockholm, Sweden; 4 Division of Physiological Chemistry II, Department of Medical Biochemistry and Biophysics, Karolinska Institutet, 17165 Stockholm, Sweden; University of Southampton School of Medicine, UNITED KINGDOM

## Abstract

An important step in the production of inflammatory mediators of the leukotriene family is the Ca^2+^ mediated recruitment of 5 Lipoxygenase (5LO) to nuclear membranes. To study this reaction *in vitro*, the natural membrane mimicking environment of nanodiscs was used. Nanodiscs with 10.5 nm inner diameter were made with the lipid POPC and membrane scaffolding protein MSP1E3D1. Monomeric and dimeric 5LO were investigated. Monomeric 5LO mixed with Ca^2+^ and nanodiscs are shown to form stable complexes that 1) produce the expected leukotriene products from arachidonic acid and 2) can be, for the first time, visualised by native gel electrophoresis and negative stain transmission electron microscopy and 3) show a highest ratio of two 5LO per nanodisc. We interpret this as one 5LO on each side of the disc. The dimer of 5LO is visualised by negative stain transmission electron microscopy and is shown to not bind to nanodiscs. This study shows the advantages of nanodiscs to obtain basic structural information as well as functional information of a complex between a monotopic membrane protein and the membrane.

## Introduction

Inflammation is one of the innate defense mechanisms exerted by the human body for protection and to initiate the healing process. Chronic inflammatory reactions are involved in several disease conditions as diverse as atherosclerosis, asthma, rheumatoid arthritis, cancer and obesity. Leukotrienes (LT:s, see abbreviations list), one of several groups of pro-inflammatory lipid mediators derived from arachidonic acid, are involved in inflammatory diseases. 5-lipoxygenase initializes the leukotriene biosynthesis cascade by oxygenating arachidonic acid, released by cytosolic phospholipase A_2_ (cPLA_2_) from nuclear membranes. Arachidonic acid is converted into 5HPETE and further dehydrated to the allylic epoxide Leukotriene A_4_ (LTA_4_) by 5LO. LTA_4_ is then converted to LTB_4_ by the cytosolic protein LTA_4_ hydrolase or alternatively acts as substrate for an integral membrane protein, LTC_4_ synthase, that forms LTC_4_, a precursor to LTD_4_ and LTE_4_. The final products of the leukotriene cascade are LTB_4_, LTC_4_, LTD_4_, and LTE_4_ **[1,2]**.

The recently published structure of human 5LO (PDB ID: 3O8Y, 78 kDa) shows the expected N-terminal β-sandwich (residues 1–114) and a larger non-heme iron containing C-terminal catalytic domain (residues 121–673), connected by an inter-domain peptide **[3]**. The structure resembles a cylinder 9 nm long and 4.5 nm in diameter. Several factors influence 5LO activity via the β-sandwich, the most important of which is binding of Ca^2+^ that leads to association with the nuclear membrane and increased enzyme activity **[4,5,6,7]**. The binding of 5LO to nuclear membrane does not depend on interactions with other proteins, although the 5LO scaffold proteins FLAP and CLP have been shown to promote association of 5LO with the nucleus, leading to increased cellular 5LO activity **[8,9,10]**. The CLP which binds to 5LO, can replace and complement membranes as a scaffold for Ca^2+^ -induced 5LO activity *in vitro* **[11]**. For all lipoxygenases to become active, it is required that the prosthetic non-heme iron is oxidized to the ferric form, by lipid-hydroperoxides **[12]**. On the other hand, oxidizing conditions may also lead to inactivation and dimerisation of 5LO via cystein bridges **[13]**.

Ca^2+^ mediated binding to the nuclear membrane of 5LO and its activation has been successfully studied *in vitro* using traditional methods like liposomes **[14]**, detergent micelles **[15]** and membrane preparations **[16]**. However, these methods have drawbacks such as the large size of liposomes and inaccessibility to the liposome interior. For structural studies of 5LO binding to the membrane by transmission electron microscopy (TEM), liposomes could possibly be used [17,18,19], however we have chosen another membrane platform, so called nanodiscs (ND). Nanodiscs self-assemble from two molecules of membrane scaffolding protein (MSP) and phospholipids **[20]**. The lipid molecules are non-covalently assembled like a bilayer and two molecules of MSP wrap around the hydrophobic lipid alkyl chains in a belt-like manner forming a disc (diameter 10–20 nm) shaped structure [21]. The nanodisc can be made as an “empty” nanodisc which can be used only to mimic the membrane but it can also self-assemble with membrane proteins [22].

Previously, large unilamellar liposomes were used to show that cationic lipids have a stimulatory effect and negatively charged lipids have an inhibitory effect on 5LO activity [23] compared to the zwitter-ionic phosphatidyl choline (PC) head group. It was argued, however, that the nature and composition of phospholipid head groups is not the main reason for the translocation preferentially to the nuclear membranes [23]. Rather, a high membrane fluidity due to enrichment in lipids with a high number of *cis* unsaturated bonds in the sn-2 acyl chain proved more important. It was shown that the use of POPC, that contains one unsaturated bond, compared to the use of a saturated lipid (DPPC) resulted in the largest increase in activity of 5LO with some further increases in activity with increased number of unsaturated bonds in the sn-2 acyl chain [24]. Hence, for these initial studies of 5LO binding to nanodiscs we chose POPC as the bilayer in the nanodiscs.

In this paper we exploited the nanodisc property as a membrane mimic to study binding and activation of human recombinant 5LO. Both monomer and dimer 5LO were investigated. Ca^2+^ mediated binding of monomeric 5LO on nanodiscs is visualized by both native gel electrophoresis and negative stain electron microscopy. Activity assays show that monomeric 5LO is active and stable on the nanodiscs. Dimeric 5LO could be visualized by negative stain electron microscopy and are shown to not bind on nanodiscs. The use of nanodiscs made these experiments possible and form the basis for future structural studies of the 5LO-Ca^2+^ -ND complex by high resolution cryoEM to shed light on the mechanisms of 5LO activation.

The abbreviations used are: AA, arachidonic acid; CLP, Coactosin like protein; FLAP, Five lipoxygenase activating protein; MSP, Membrane scaffolding protein; DPPC, di-palmitoyl-sn-glycero-3-phosphocholine; 5HPETE, 5-(S)-hydroperoxy-6-trans-8,11,14-cis-eicosatetraenoic acid; 5HETE; 5-(S)-hydroxy-6-trans-8,11,14-cis-eicosatetraenoic acid; 13-(S)-HPODE, 13-(S)-hydroperoxy-9-cis-11-trans-octadecadienoic acid; 5LO, 5-lipoxygenase; LT, leukotriene; LTA4, 5(S)-trans-5,6-oxido-7,9-trans-11,14-cis-eicosatetraenoic acid; ND, Nanodisc; PC, phosphatidylcholine; POPC, 1-palmitoyl-2-oleoyl-sn-glycero-3-phosphocholine; PTA, Phosphotungstic acid; SEC, Size Exclusion Chromatography; TEM, transmission electron microscopy; UF, Uranyl formate.

## Materials and Methods

### Chemicals

If not otherwise stated, chemicals are from Sigma-Aldrich Co.

### Expression and purification of 5LO and MSP1E3D1

Human 5LO was expressed in *E*. *coli* BL21-(DE3) (NEB) transformed with pT3-5LO [25] and purified with ATP Agarose column (Sigma-Aldrich Co.) followed by gel filtration [24]. As an outline, the post induction cells were resuspended in lysis buffer (100 mM Tris-HCl pH 7.5, 100 mM NaCl, 2 mM EDTA, 1mM FeSO_4_, 2 mM TCEP) with protease cocktail inhibitor and lysozyme 0.5mg/ml) followed by sonication (5 x 15s). After clarifying the lysed cells, the supernatant was subjected to ammonium sulfate precipitation and the precipitate (30–60% saturation) was resuspended in lysis buffer. The sample was applied to an ATP agarose column and the recombinant 5LO was eluted using 20mM ATP in lysis buffer with 10 μM FeSO_4_ and 20 μg/ml catalase [26]. The buffer was changed to 20 mM Tris pH 7.5, 100 mM NaCl, 2 mM EDTA, 1mM FeSO_4_, 2 mM TCEP, 20 μg/ml catalase. The concentration of 5LO in elutaes was determined by Bradford assay **[27]**. Also MSP1E3D1 (with a histidine tag as well as a TEV protease cleavage site, from Addgene, MA, USA) [21] was expressed in *E*. *coli* (BL21-DE3). The concentration of purified MSP1E3D1 was determined by the absorbance at 280 nm (ε = 29910cm^-1^M^-1^).

### Preparation of ND

An appropriate amount of chloroform dissolved POPC (Avanti polar lipids) was dried under nitrogen followed by overnight removal of residual chloroform in a vacuum desiccator. POPC was resuspended in MSP standard buffer (25 mM Tris-HCl pH 7.5, 100 mM NaCl, 0.5 mM EDTA) supplemented with sodium cholate (Anatrace, USA). (POPC: sodium cholate molar ratio of 1:2). His-tagged MSP1E3D1 was added to the re-suspended lipids in a molar ratio of 1:130 (MSP1E3D1: POPC) and incubated on ice for one hour. Biobeads (Biorad) 0.5 mg/ml was added to initiate the self assembly process and incubated in a rotary incubator for 16 hours at 4°C **[28]**. After clarifying the supernatant by centrifugation at 13000 x g for 10 min at 4°C, the sample was purified by gelfiltration using an Agilent Bio SEC-5 column, equilibrated with MSP standard buffer. The concentration of nanodiscs was determined by the absorbance at 280 nm using the molar extinction coefficient of MSP1E3D1 (ε = 29910cm^-1^M^-1^).

### Electrophoresis and protein immunoblots

5LO and MSP1E3D1 preparations were analyzed by SDS page (4–20% Tris-glycine gel, Invitrogen). SDS PAGE was performed in 12% Tris glycine gel (in-house prepared). All native PAGEs are performed in 4–16% Bis- Tris gel (NativePAGE™ Novex, Invitrogen), with small changes in buffers explained later. Briefly, samples were mixed with 4X Native PAGE Sample Buffer (Invitrogen) and loaded onto Bis-Tris gel. Blue native page was performed by using light cathode buffer (NativePAGE™ Running Buffer with 0.001% Coomassie G-250) in the cathode tank and NativePAGE™ Running Buffer as anode buffer. Samples ran on gel until the Coomassie front reached the end of the gel at a constant 150 V. Normal native PAGE was performed by using NativePAGE™ Running Buffer as both cathode and anode buffer. Ca^2+^ native PAGE was performed in two steps; an overnight equilibration run was performed with NativePAGE™ Running Buffer containing 1mM CaCl_2_ and 5 mM β-mercaptoethanol at 30 V at 4°C. The separation run was performed the next day at 150 V, 4°C with same buffer. All the gels were stained using Coomassie Brilliant Blue R-250. For immunoblotting proteins were transferred (by electrophoresis) from SDS PAGE gels to nitrocellulose membrane. Rabbit polyclonal antibody for 5LO from Cayman chemicals was used for identification. Positive signals were visualized with ONE-HOUR Western™ Detection System (Genscript) using 3,3,5,5-Tetramethylbenzidine (Sigma) as substrate.

### Negative Stain Electron Microscopy and class-averaging

In-house carbon coated copper grids (400 mesh, TedPella) were glow-discharged (Baltec) to render them hydrophilic before adsorption of 3.5 μl of analytes for 30 seconds. Surplus solution was blotted off on filterpaper. Immediately, the grid was stained with a drop of either 2% phosphotungstic acid (PTA) at pH 7.4 or 1% Uranyl Formate (UF) for 30 seconds, then surplus solution was blotted off and the grid allowed to dry. The grids were analyzed in a calibrated Jeol 2100F TEM (200keV accelerating voltage) and images were acquired by a 4K x 4K CCD camera (Tiez Video and Imaging Processing System GmbH, Germany) at a magnification of 69500. The pixel size of the CCD camera is 15 μm which gives a corresponding value of 2.16 Å on the specimen level for the EM images. For the collection of negative stain data, new grids as just described, were made on at least three different days and for each day, fresh sample incubations were made before the negative stain was applied.

Processing of the images was done by the software EMAN2 [29]. Micrographs obtained were manually sorted to have a good signal to noise level and particles were picked manually. This is followed by 2D-class averaging [30]. The principle of 2D class-averaging is to generate a small set of representative class-averages from a large set of boxed out particles by using an iterative reference-free classification algorithm. This process reduces the noise levels in the results and makes the views of particles better observed. The initial iteration starts with some invariant-based guesses and the clustering of raw particles is based on the differences among particles detected by the less-precise MSA (multivariate statistical analysis) method. This therefore makes the initial results (class-averages) lacking in correctness. Then the initial class-averages are aligned to each other and sorted by similarities. By comparing with the aligned class-averages generated from the last iteration, raw particles are re-allocated and clustered into new class-averages in the new iteration. During all the iterations, particles with similar views are slowly clustered into the same classes, and this means the class-averages become more and more representative. Ideally, as a result, the final output contains a certain number of particle class-averages and each of them is obtained from averaging a series of similar particles. However, practically the particles within the same class may not be so homogeneous, and in this case, the features of those particles with different views would be averaged out during the class-averaging [29,30].

### UV spectroscopy

Initial rates of 5LO were measured in a UV—spectrophotometer (Tecan Infinite M200 Pro, Tecan Group, Ltd, Switzerland). In a 100 μl quartz cuvette (1 cm), 50 μL assay buffer (50 mM Tris-HCl pH 7.5 and 2 mM EDTA) was mixed with 40 μl substrate mix. The latter contains Tris-HCl pH 7.5, AA (Avanti polar lipids Inc, AL, USA), 13-(S)-HPODE (Cayman Chemical Company, MI, USA), MgCl_2_ with or without CaCl_2_ and was sonicated for one minute at room temperature. Three pairs of substrate mix were made with and without Ca^2+^: 1) no membranes, 2) ND or 3) L-α PC from egg (Avanti Polar lipids Inc, AL, USA). The final concentrations in the reaction were 77.4 mM Tris-HCl pH 7.5, 1.2 mM EDTA, 20 μM AA, 2 μM 13-(S)-HPODE, 6.2 mM MgCl_2_, with or without 1.3 mM CaCl_2_, 10 μg/ml ND or 10 μg/ml L-α PC liposomes. The reaction was started by adding 10 μl of 5LO (0.5μg) in assay buffer to the cuvette. Equilibrium concentrations of free and complexed divalent ions were calculated with the software **[31]** see results section. The product conjugated dienes (ε = 23000 M^-1^cm^-1^) was monitored at 235 nm for five minutes at room temperature [11]. ATP was omitted due to its strong absorbance at the product wavelength.

### LC-MS/MS

The amount of LTA_4_ formed by 5LO was analyzed by determining the nonenzymatic hydrolysis products (6-trans-LTB_4_ and 12-epi-6-trans-LTB_4_) by LC-MS/MS. The assay procedure was essentially followed from Rakonjac et al, 2006 [11] with some modifications. In an eppendorf tube, assay mix were prepared by mixing 50 μL assay buffer (50 mM Tris-HCl pH 7.5 and 2 mM EDTA) with 40 μl substrate mix. The latter contains Tris-HCl pH 7.5, AA, 13-(S)-HPODE, MgCl_2_, ATP, with or without CaCl_2_ and was sonicated for one minute at room temperature. Three pairs of substrate mix were made with and without Ca^2+^: 1) No membranes, 2) ND or 3) L-α PC from egg. The reaction was started by adding 10 μl 5LO (0.2 μg) in assay buffer to the assay mix (total volume 100 μl) and incubated for 10 minutes at room temperature. The final concentration of components in the mixture were 77.4 mM Tris-HCl pH 7.5, 1.2 mM EDTA, 50 μM AA, 5 μM 13-(S)-HPODE, 6.2 mM MgCl_2_, 4.9 mM ATP with or without 1.3 mM CaCl_2_, 25 μg/ml ND or 25 μg/ml L-α PC liposomes. Due to the complexing agents, EDTA and ATP, equilibrium concentrations of free and complexed divalent ions were calculated with the software BAD [31], see results section. The enzyme activity was stopped with 400 μl of cold stop solution (Acetonitrile:Water:Acetic acid in a ratio of 60:40:0.2: v/v) [11].

LC–MS/MS analysis was performed on a Waters 2795 HPLC (Waters Corporation, MA, USA) coupled to a triple quadrupole mass spectrometer (Acquity TQ Detector, Waters Corp.). An aliquot of 15 μl was injected onto a Synergi Hydro-RP column (100 mm × 2 mm i.d., 2.5 μm particle size and 100 Å pore size, Phenomenex, CA, USA). A gradient was used starting at 35% mobile phase A (MilliQ water, Merck Millipore KGaA, Germany) and 65% mobile phase B (MeOH, 0.05% formic acid) for 34 min, then increased to 95% B over 1 min and stayed at 95%B for 15min before re-equilibration at 65%B for 14 min. The flow rate was 100 μl/min. The analytes were detected in multiple reaction monitoring (MRM) in negative mode utilizing the following transitions: m/z 335>196 and m/z 335>317 for LTB_4_ and its isomers (6-trans-LTB_4_ and 12-epi-6-trans-LTB_4_). All analyzed using collision energy of 20V and cone voltage 30V. Analysis of the MRM data was carried out by MassLynx software, version 4.1 (Waters Corp.), using external standard calibration of LTB_4_.

### Statistical analysis

All micrographs from TEM and polyacrylamide gel pictures are representative for a minimum of three independent experiments. A value of P<0.05 was considered significant according to the criteria of Student’s *t* test. The activity assay results are expressed as mean±SEM of *n*.

## Results

### Calcium ions induced binding of 5LO on nanodiscs

**Fig 1:**
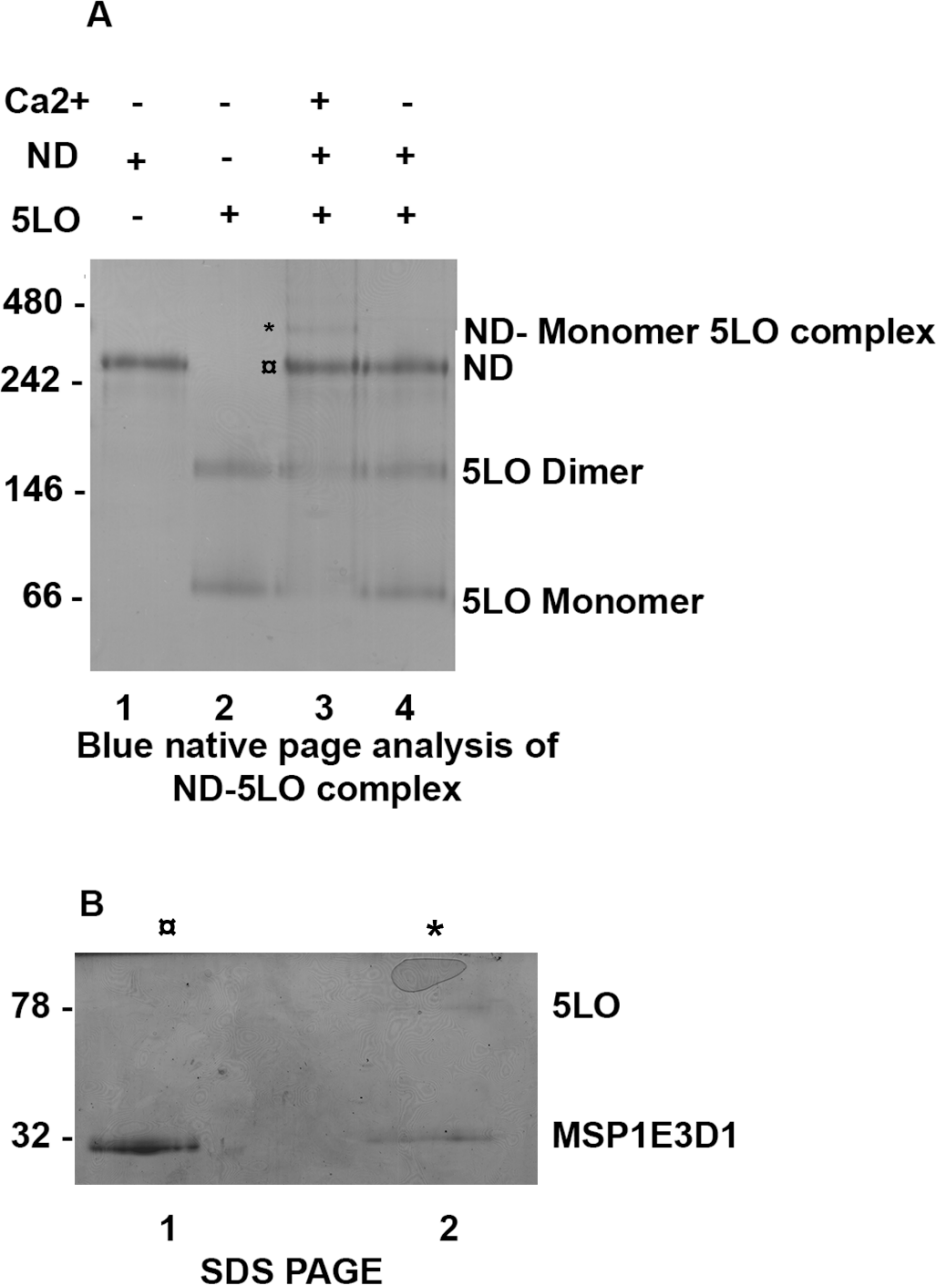
5LO can bind to nanodiscs in the presence of Ca^2+^. (A) Blue native PAGE (4–16% Bis Tris gel) analysis of ND-5LO complexes formed after incubations of ND and 5LO with and without 1 mM Ca^2+^ present. Lane 1, purified ND. Lane 2, purified 5LO. Lane 3, sample from incubation of 5LO, ND and Ca^2+^. Each lane is loaded with 1.5 μg of protein. The bands at ≈340 kDa (*) and ≈260 kDa (¤) were excised and subjected to SDS-PAGE. Lane 4, sample from incubation of 5LO and ND without Ca^2+^. (B) SDS PAGE (12% Tris-glycine gel) denaturing analysis of the two bands excised from BN-PAGE lane 3. Both excised bands contain MSP1E3D1 (32 kDa) but only the ≈340 kDa (*) band contain 5LO (78 kDa).

Nanodiscs were prepared using the phospholipid POPC and MSP1E3D1. This version of membrane scaffolding protein can form a disc with inner diameter ~ 10.5 nm containing about 260 molecules of POPC **[32]**. The calculated molecular weight is about 260 kDa and bilayer surface area 8900 Å^2^. The ND was purified by SEC (data not shown) and homogeneity was analyzed by BN-PAGE (Fig 1A, Lane 1) showing only one band as expected at around 260 kDa.

Aliquots of ND (0.6 μM) and 5LO (0.6 μM) were mixed and incubated with or without 1 mM Ca^2+^ present in the buffer and binding was determined by BN-PAGE. In the presence of Ca^2+^ an additional band appeared above the ND band, corresponding to a complex containing one ND and one 5LO (78 kDa) (expected molecular weight approx. 340 kDa, see Fig 1A, Lane 3). To confirm the presence of 5LO in the top band (at 340 kDa (*)), it was excised from the gel and subjected to SDS PAGE (Fig 1B). Two bands appeared, corresponding to 5LO (top) and MSP1E3D1 (bottom) (Fig 1B, Lane 2 (*)) whereas the cutout from the ND band (Fig 1A, lane 3, (¤)) shows only the MSP1E3D1 protein (Fig 1B, Lane 1, (¤)). It should be noted that the bands in lane 3 reflects the instability of the sample loaded. The 5LO-ND complex gradually falls apart during the calcium-free gel-run. The 340 kDa band is faint, and both ND and 5LO monomer bands are clearly present in lane 3. Nevertheless, the band at 340 kDa contains 5LO (Fig 1B, Lane 2). Although the Ca^2+^ binding is reversible, the Ca^2+^ binding to 5LO has a K_D_ of only 6 μM [33] and as no precautions to remove Ca^2+^ completely during the gel-run were made, the calcium levels may be sufficient for some complexes to remain. In summary, 5LO can form complexes with nanodiscs in the presence of Ca^2+^ ions, as expected.

### Size exclusion chromatography of 5LO protein: Separation of dimers from monomers

**Fig 2:**
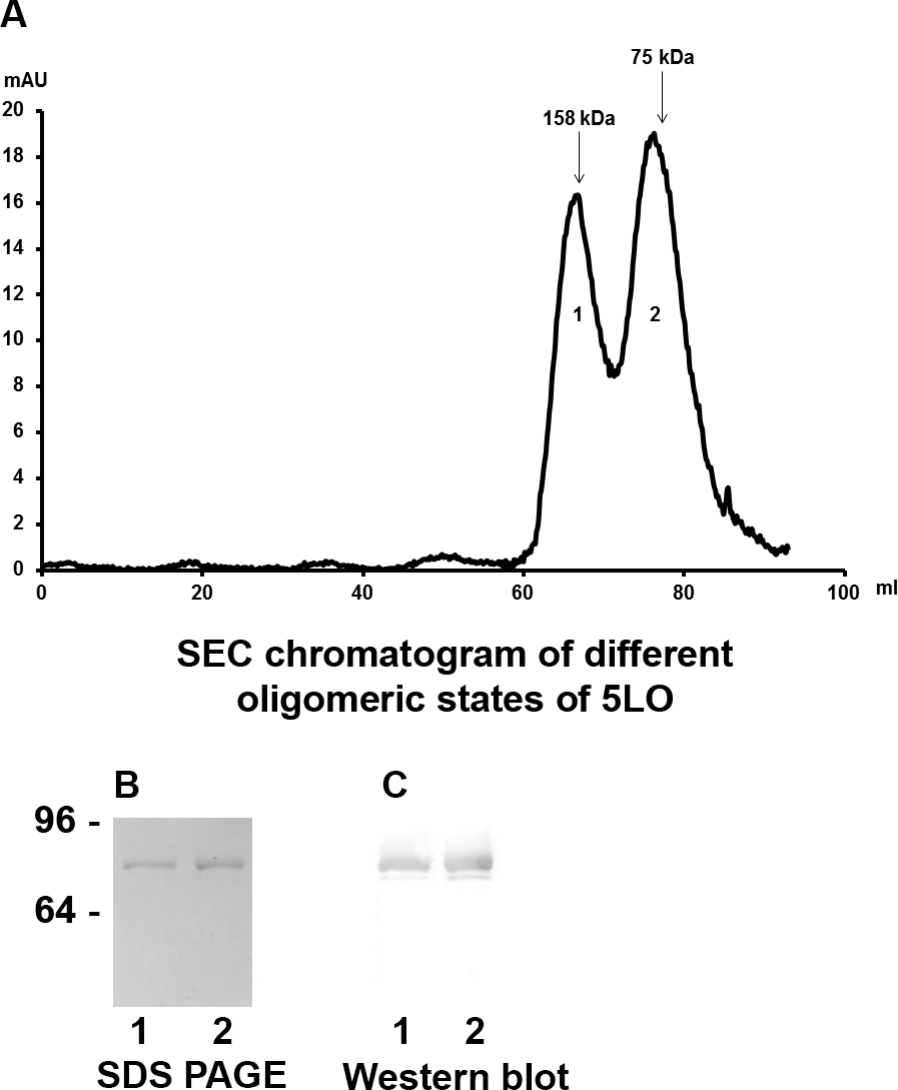
Recombinant 5LO exist as monomer as well as dimer. (A), Size exclusion chromatography of 5LO. Arrows indicate elution of standard proteins: Aldolase (158 kDa), Conalbumin (75 kDa). Peak 1 (retention volume 66 ml) corresponds to 5LO dimer. Peak 2 (retention volume 78 ml) corresponds to 5LO monomer. (B and C) SDS-PAGE of material in SEC peaks (lanes 1, from peak1; lanes 2, from peak 2) (B) Coomassie stained gel. (C) Western blot with 5LO antibody.

In the BN-PAGE analysis (Fig 1) all samples containing 5LO showed an additional band above monomeric 5LO (Fig 1A lane 2,3,4) with an approximate molecular weight 158 kDa corresponding to dimeric 5LO. To determine the presence of oligomers in our 5LO preparation, size exclusion chromatography (SEC) was performed. Two peaks at retention volumes of 66 and 76 ml (Fig 2A, peaks labelled 1 and 2) were observed. The material in the two peaks was concentrated and analysed by western blot (Fig 2C) and by staining of the SDS gel (Fig 2B). Both peaks 1 and 2 contain 5LO. The 5LO dimer was not stable under reducing SDS-PAGE conditions The monomeric and dimeric 5LO preparations were used for further experiments as described below.

### Monomeric 5LO binds on nanodiscs: the highest ratio is two 5LO per nanodisc

**Fig 3:**
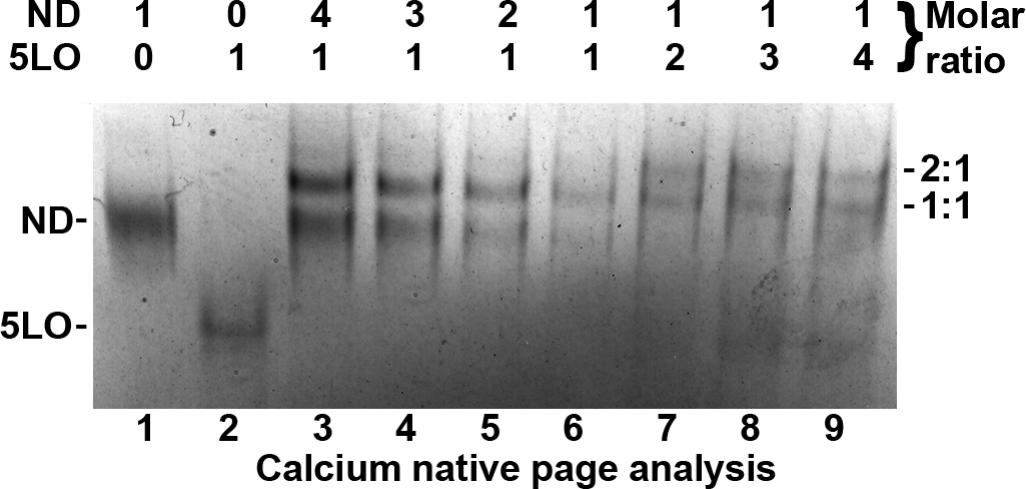
Maximum of two 5LO can bind to one ND. Calcium native PAGE analysis of ND and 5LO incubations in presence of Ca^2+^, with stoichiometries ranging from 4:1 to 0.25:1 (ND:5LO). From lanes 3 to 5, the concentration of ND is decreasing with constant 5LO. Lane 6, 5LO and ND are in equimolar ratio (0.22 μM). From lanes 7 to 9, the concentration of 5LO is increasing with constant ND. See results for further details.

To determine the stoichiometry of binding of 5LO to ND, a titration experiment was performed where monomeric 5LO (prepared by SEC) and ND were incubated at different molar ratios (1:4 to 4:1) in presence of Ca^2+^. The samples were analyzed by Ca^2+^ native PAGE to stabilize the Ca^2+^ mediated interactions, and under reducing conditions to prevent dimerisation of 5LO (Fig 3). For lane 6 equimolar concentrations (0.22 μM) of 5LO and ND were mixed. The major band observed corresponds to the 1:1 complex formed during incubation. A small amount of unbound nanodiscs can also be observed in lane 6. In lane 3, ND was in 4 times excess, in lane 4 ND was in 3 times excess, and in lane 5, ND was in 2 times excess, in relation to 5LO. Excess ND did not seem to change the number of 5LO binding per ND; the 1:1 band, as well as free ND, is present in lanes 3–5. In lane 7 5LO was in 2 times excess, in lane 8 5LO was in 3 times excess, and in lane 9, 5LO was in 4 times excess, inrelation to ND. The presence of the apparent 2:1 band in lanes 7–9 indicates that two 5LO can bind to one ND, but not more than this. In lanes 7–9 also the 1:1 complex and free 5LO can be observed, but no free ND. The most likely explanation is that each of the nanodisc lipid surfaces can accommodate maximally one 5LO molecule.

### TEM analysis of monomer 5LO-ND

**Fig 4:**
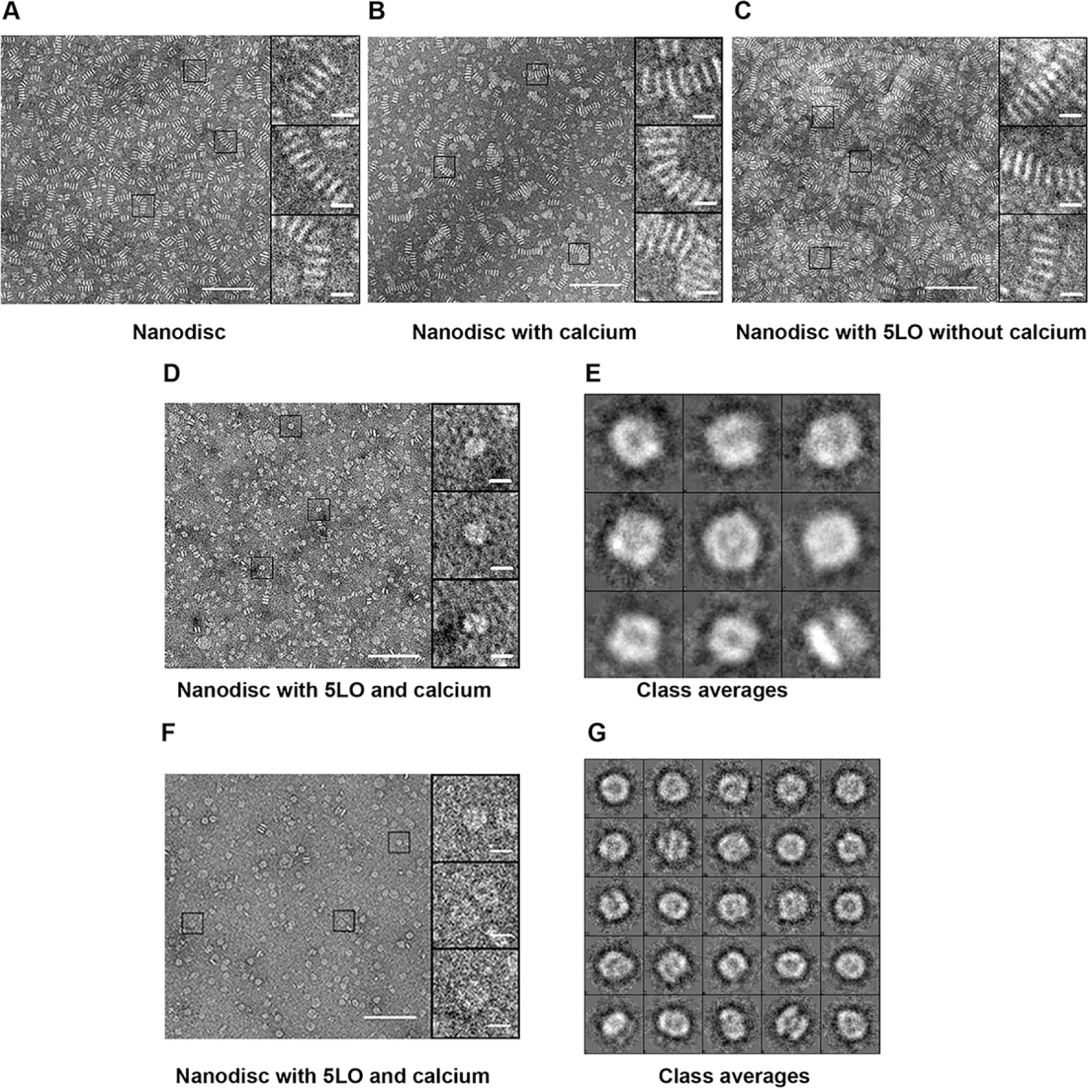
Both indirect and direct visualization of the effect of calcium on interaction of monomeric 5LO with ND, by negative stain electron microscopy. (A), ND showing stack formation induced by PTA stain, homogeneous size and the rigid structure of ND. (B), the ND preparation as in A but in the presence of Ca^2+^ shows the same amount of stacking as in A. (C), PTA-stained samples of equimolar concentrations of ND incubated with 5LO in calcium-free buffer, clearly showing stack formation, which signifies lack of interaction between 5LO and ND. (D), PTA stained samples of equimolar concentrations (0.8 μM) of ND incubated with 5LO in the presence of 1 mM Ca^2+^, showing very few stacks. A lack of PTA-induced stacking formation, signifies the interaction between 5LO and ND. The magnifications on the right in are representatives of the majority of the particles found in the corresponding image. (E), Class averages of 5LO-ND-complexes stained with PTA. Five images were used (including the one in D) to box 377 particles. Box-size is 20.8 nm. Scale bars are 10 nm and 100 nm respectively. (F), PTA stained samples of ND (0.8 μM) incubated with 5LO (1.6 μM) in the presence of 1 mM Ca^2+^, showing even fewer stacks. Most particles are larger and have different shapes compared to the 1:1 ratio shown in (D). (G), Class averages of 5LO-ND-complexes stained with PTA. Thirty six images were used to box 573 particles. Box-size is 26.6 nm. Scale bars are 10 nm and 100 nm respectively.

In the process of negative staining, the contents in a small volume of the biological sample is adsorbed onto a transparent carbon support and excess liquid removed. A few microliters of a heavy metal salt is applied and the specimen allowed to dry. Fine grains of salt microcrystals are thereby produced and cover the particles in crevices and around the particle (Figure B in S1 File) [34]. The image formed looks like a photographic negative, i.e. white objects on a dark background. Of note from this description of negative staining is that chemical interaction with the sample should not occur (*e*.*g*. for uranyl salts). However, in some rare cases this does occur as for the negative stain phosphotungstic acid (PTA) with lipid bilayers forming “rouleaux”or “stacks” (Figure C in S1 File [35]), an effect that we decided to use to our advantage for nanodisc lipid bilayers. Hence, there are two aims with PTA staining of our samples: 1) direct observation of complexes formed. Here we can collect particles (“boxing”) from the TEM images, classify and average these to produce direct views of the complexes formed from different angles [30]. 2) also indirectly show that 5LO has bound to ND. The stacking due to the PTA should not be possible if a protein is bound on and protects the surface of the ND bilayer. With respect to aim 2, the analysis of stacking in PTA-stained samples: For ND, the sample was homogeneous (Fig 4A) and stacked as expected [35]. Incubating nanodiscs with Ca^2+^ does not prevent stacking (Fig 4B). In the sample of monomeric 5LO and ND incubated without calcium, the PTA induced stacking event is still present (Fig 4C). Thus, in the absence of calcium ions, 5LO remains unbound. This means the ND can bind each other in stacks (Figure C in S1 File). However, in Fig 4D, using the sample containing the complex of 5LO-ND formed in the presence of calcium (Figure E in S1 File), stacking was almost completely absent. This shows that the calcium ions induce 5LO binding on nanodiscs and as a consequence, steric hindrance prevents the ND to stack.

With respect to aim 1, what kind of particles, viewed from different angles, can be expected to be observed? 5LO is nearly cylindrical with a long dimension of 9 nm and the diameter 4.5 nm [3]. The ND dimensions are in fact similar to 5LO as the outer diameter is around 12.5 nm. The thickness of a POPC bilayer was measured in a solution in unilamellar or multilamellar liposomes to be about 4nm at room temperature [36]. A close to parallel orientation of the long dimension of the 5LO cylinder and the lipid bilayer was proposed [24]. Therefore, vieweing a complex of one 5LO embedded on a nanodisc from the side (Figure E4 in S1 File) would be expected to look as a “bipartite” object. Such particles can be observed in Fig 4D where three magnifications are shown to the right. Particles in images like the one in Fig 4D were boxed and compared to each other with the aim to assign each particle to similar ones, grouped in a class. The ideally identical particles within each class are averaged to highlight details and reduce noise. They should also represent different viewing angles of the particle [30]. Fig 4E shows class-averages of the complex formed between 5LO and ND incubated at a 1:1 ratio in the presence of Ca^2+^. The lower row of class-averages could show side views of a bipartite object as proposed above and as in figures E3 and E4 in S1 File. The other class-averages could be more tilted views or show top views.

For 5LO and ND mixed at a ratio of 1:1 the complexes are around 10 nm (Fig 4D and 4E). However, incubation with an excess of 5LO to ND in the presence of Ca^2+^, show particles larger than 10 nm (Fig 4F). In fact, according to the results in Fig 3, lanes 7–9 representing an excess of 5LO to ND, we should expect a mix of particles containing one 5LO per nanodisc (ca 10 nm) and two 5LO per nanodisc (> 10nm). Furthermore, how would complexes of two 5LO and one ND assemble? With one 5LO on each side of the nanodisc some kind of “tripartite” particle could be expected (Figures F2 and F3 in S1 File). However, the tryptophans and loops shown to mediate the binding of 5LO to the membrane are located in the N-terminal β-sandwich. Therefore it cannot be ruled out that two 5LO can bind on the same side of a nanodisc (Figure F4 in S1 File).

In an attempt to clarify this, images were taken, e.g. the one in Fig 4F and class-averaging was performed, shown in Fig 4G. The number of classes is higher than in Fig 4E, due to the presence of both 1:1 and 2:1 complexes, all viewed from different angles (cf. Figures E and F in S1 File). With the numbering as class 0 in the lower left and 24 in the top right corner, several class averages seems to show “tripartite” objects, e.g. 6 and 16 formed by a nanodisc with one 5LO on each side of the bilayer viewed from the side (Figure F2 in S1 File). We speculate that class-average number 3 (Fig 4G) shows two 5LO on the same side of the nanodisc. A “bipartite” view of a 1:1 complex could be class-average 7 in Fig 4G.

As mentioned earlier, negative staining should not interact chemically, but cover particles that are homogenous and evenly spread on the support. Furthermore, the particles should adsorb to the support randomly and not in just one preferred orientation. As shown by Zhang et al [35], the negative staining by a uranium salt prevents phospholipid bilayers to stack. Their result show only one preferred view, “top views” (Figure A1 in S1 File). Compared to PTA, the formate salt has a smaller grain size. Therefore a somewhat better resolution may be obtained although UF is more difficult to work with. An attempt to obtain more detail and to verify the presence of the 5LO-ND complexes also in the uranyl salt UF was made (Figure G in S1 File). The sizes of the particles were to a large extent more than 10 nm and similarities to the “tripartite” complexes stained by PTA was found indicating the presence of the 5LO-ND complex also after staining by UF (Figure G2 in S1 File).

### The activity of monomeric 5LO is comparable on nanodiscs *vs* liposomes

Next we compared the effect of two different membrane mimicking enivronments, ND or liposomes, on 5LO activity (in the presence or absence of calcium). From an estimation that the two MSP1E3D1 encircles 260 molecules of POPC [32] the total concentration of phospholipid was calculated for the ND preparation. For liposomes we used L-α-PC at the same total lipid concentration as for the nanodiscs.

Initial rates and 5HETE formation: A major 5LO product is 5HPETE which is concomitantly non-enzymatically reduced to the hydroxyacid 5HETE. This reaction was followed by UV spectroscopy at 235 nm, and the initial rate of product formation calculated (V_init_, Table 1). Also the final amount of product formed after the 5 min reaction is shown (Final Product, Table 1). These measurements were performed in the presence of around 5 mM free Mg^2+^ and an AA concentration of 0.02 mM. In the presence of 10 μg/ml phospholipids we find an initial activity and amount of final product that is relatively low. This corresponds well with earlier results showing that at low concentrations of AA and PC, Mg^2+^ has a negligible 5LO activating effect. When also Ca^2+^ (0.35 mM) was added there was a strong increase in 5LO activity (see Table 1): from 0.14 to 1.87 μmol/mg/min in presence of nanodiscs; and 2) from 0.06 to 1.35 μmol/mg/min in presence of PC. Also the amounts of 5HPETE formed after 5 min showed strong Ca^2+^ dependent increases (see Final Product, Table 1). In the absence of lipids the initial activity was low as expected, 0.12 and 0.20 μmol/mg/min.

**Table 1:** 5LO initial velocity and product formation. Initial velocities (column V_init_) and total 5-HPETE/5-HETE formation (column Final Product) were determined by UV spectroscopy at 235 nm, during 5 min incubations with different activating factors. Ratio 5LO:ND is 1.3:1. Data are mean±SEM (n = 3). LTA_4_ formation (column LT) in 10 min incubations with different activating factors was measured by LC-MS/MS. * Not detected. Data are mean±SEM(n = 2).

Assay Components	V_init_ μmol/mg per min	Final Product μmol/mg	LT μmol/mg
**5LO+AA**			
With Ca^2+^	0.20±0.02	0.70±0.08	0.69±0.04
With out Ca^2+^	0.12±0.01	0.55±0.09	0.69±0.03
**5LO+AA+ND**			
With Ca^2+^	1.87±0.12	3.60±0.10	3.29±0.25
With out Ca^2+^	0.14±0.03	0.24±0.02	0.44±0.10
**5LO+AA+PC**			
With Ca^2+^	1.35±0.06	3.32±0.13	2.41±0.05
With out Ca^2+^	0.06±0.03	0.13±0.02	n.d*

Leukotriene formation: Monomeric 5LO also catalyses the formation of LTA_4_ from 5HPETE (column LT, Table 1). LTA_4_ is unstable and degrades non-enzymatically to isomers of LTB_4_ (6-trans-LTB4 and 12-epi-6-trans-LTB4) which were measured by LC-MS/MS after 10-min incubations (column LT, Table 1). The results showed similar trend to the spectroscopy assay data for 5HPETE formation. The highest amounts of LTA_4_ was formed in the presence of both Ca^2+^ and phospholipids in the form of ND (3.29 μmol/mg), or PC (2.41 μmol/mg) (Table 1, column LT).

There is no statistically significant difference on 5LO activity mediated by either liposomes or ND in the prescence of Ca^2+^ on 5HETE/5HPETE formation (p-0.16) and in concordance formation of LTA_4_ is not significantly different (p-0.07). This shows that ND as a membrane environment is similar to liposomes. ND functions as well as vesicles and has a advantage of having a very small, defined and stable environment. On looking to activity of 5LO in the prescence of liposomes or ND but without Ca^2+^, liposomes showed a lower activity than nanodiscs, statistically significant, for both 5HETE/5HPETE formation (p-0.02) and LTA_4_ formation (p-0.05).

### Dimerisation of 5LO prevents binding to ND and inactivates 5LO

**Fig 5:**
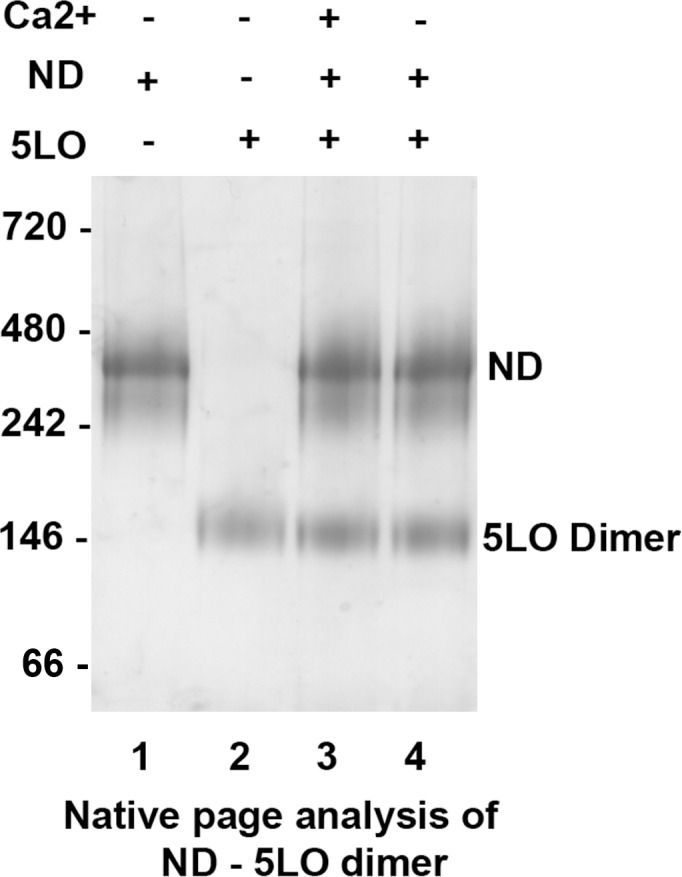
The dimeric 5LO does not bind to nanodiscs or separate into monomeric 5LO. Native PAGE analysis of purified ND (Lane 1) and purified dimer 5LO (Lane 2). Dimeric 5LO incubated with ND with Ca^2+^ (Lane 3) shows only bands corresponding to the dimer (158 kDa) and the ND (260 kDa). Dimer 5LO incubated with ND but without Ca^2+^ (Lane 4) shows the same bands as for lane 3 indicating that the dimer is not split into monomers by the presence of a membrane.

From the results above, it is clear that interaction of monomeric 5LO with Ca^2+^ upregulates binding to ND and enzyme activity (Figs 1, 3 and 4 and Table 1). We then turned to dimeric 5LO. The dimeric 5LO separated by SEC (Fig 2A, Peak 1) was incubated with ND at equimolar concentrations (0.6 μM), with and without 1 mM Ca^2+^ present, and analyzed by native PAGE (Fig 5). The approximate molecular weight of dimeric 5LO (156 kDa) in complex with ND would be 420 kDa, no band corresponding to this size was observed in the gel. Furthermore, separation of the dimer into monomeric 5LO is not induced either. The only other band in addition to ND is dimeric 5LO, present both with and without Ca^2+^ (Fig 5, Lane 3 and 4). It can be concluded that the presence of a membrane bilayer and calcium during the 1 hour incubation (before loading on the gel) does not promote any kind of complex formation, at least not one that remains stable during the gel-run.

Furthermore, the enzymatic activity of dimeric 5LO was assayed under the same experimental conditions as for monomeric 5LO. However, this yielded neither detectable amounts of 5HPETE/5HETE nor of LTA_4_ (Table A in S1 File). If the oxygenation of AA cannot be catalyzed the second step, the cyclization to the allylic epoxide would not be possible either. However, as dicussed later, AA may not be present in the catalytic site at all, depending on the assembly of the dimer with the catalytic sites facing each other, thereby blocking access to the active sites [13].

### Single Particle TEM analysis of dimeric 5LO

**Fig 6:**
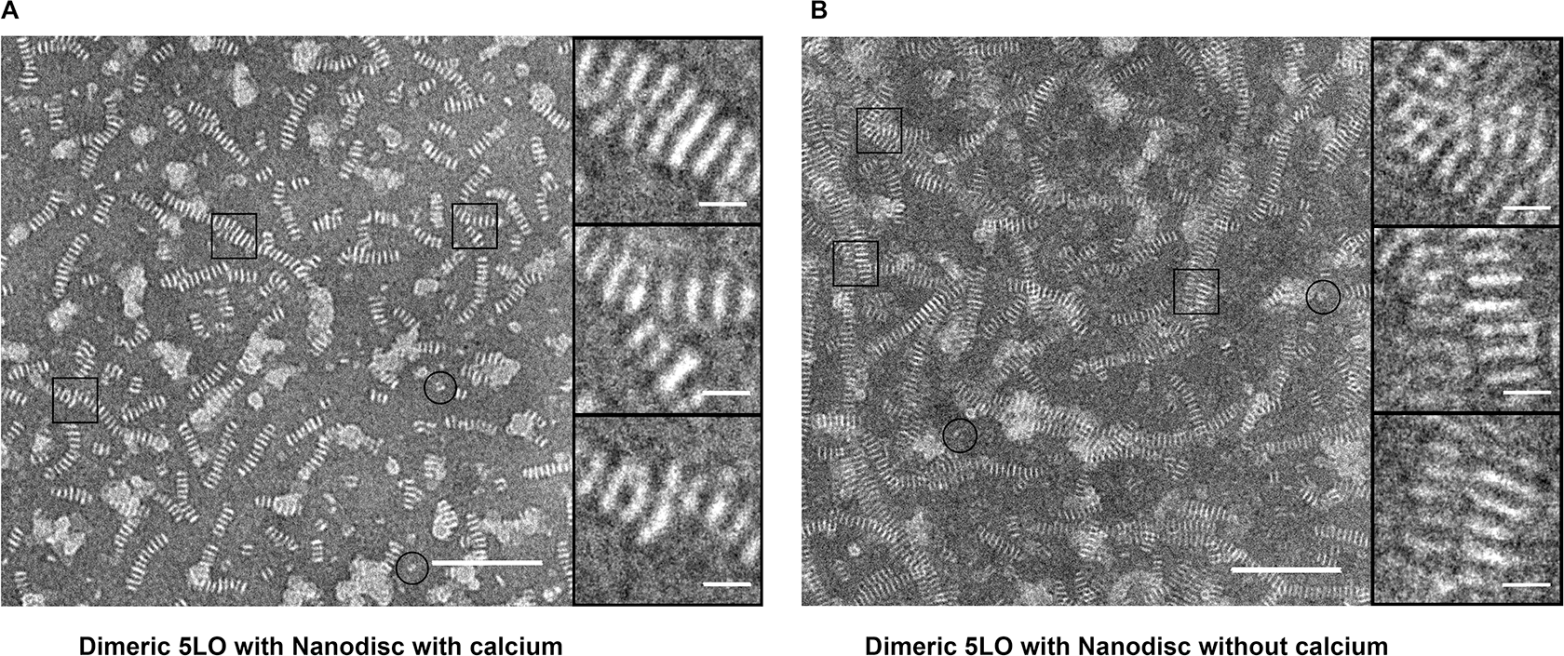
5LO dimers cannot bind on nanodiscs as shown by the extensive stacking after staining by PTA. (A) dimeric 5LO and ND incubated with Ca^2+^. Note, this preparation of nanodiscs was heterogenous and varied in the outer diameter of the discs, hence branches form (see boxes to the right). Circles show objects interpreted as unbound dimeric 5LO particles. (B) dimeric 5LO and ND incubated without Ca^2+^. Here, the preparation of nanodiscs did not vary in the outer diameter of the discs, hence no branches form. Instead, long stacks may lie on top of each other (top box) or lie parallel (middle and lower boxes). Dimeric 5LO are present (circles).

Purified dimeric 5LO was analyzed with TEM both with and without incubation with ND and with different stains. In experiments similar to those for the monomeric 5LO, we incubated the dimer 5LO with nanodiscs with or without calcium ions followed by negative staining by PTA (Fig 6A and 6B). As expected from the results shown by native page (Fig 5), the 5LO dimers did not bind to the ND neither with calcium (Fig 6A) nor without (Fig 6B). Instead, the amount of stacking observed implies that most nanodiscs are present in stacked form. Furthermore, single particles were observed of a size and shape that could represent the dimeric 5LO (circles in Fig 6A).

**Fig 7:**
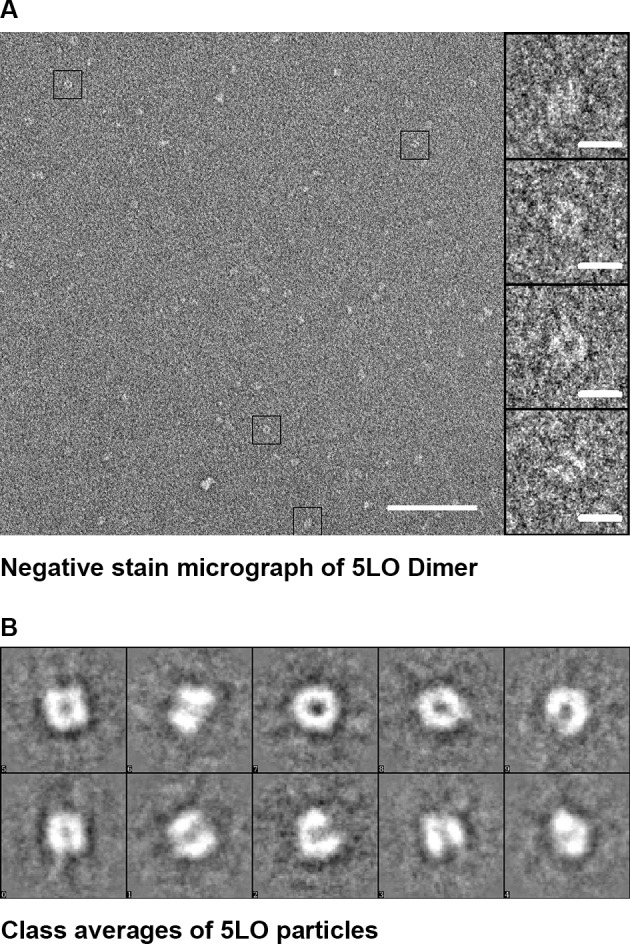
Purified 5LO dimers shown by negative stain TEM. (A), Negative stain (uranyl formate 1%) image in which four boxed dimers are shown magnified in the gallery to the right. 100 nm and 10 nm scale bars, respectively. (B), Class averages showing dimers of 5LO that conform to the proposed dimer in **[13]**. The box side length is 27.6 nm.

On the other hand, the circled particles in Fig 6 resemble two stacked nanodiscs. To establish whether this butterfly-like object would represent a 5LO dimer, purified 5LO dimers (peak 1, Fig 2A) were stained with UF for reason of its small grain size that could reveal more detail than PTA. The 5LO dimers (0.05 mg/ml) were around 10 nm and some had a butterfly appearance (Fig 7A, right, lowest box). From 39 images, a total of 695 individual particles were picked (with some examples shown in the gallery to the right in Fig 7A) and processed by the EMAN2 suite [29] into class-averages shown in Fig 7B). The class-averages indicate that the butterfly shape is a characteristic of the 5LO dimer and that no higher order oligomers forms under the conditions of the experiments.

## Discussion

The aim with the present communication was to shed light on several questions: how does 5LO bind to the membrane in our particlular system, the nanodisc? In contrast to liposomes, it actually contains a protein, the scaffolding MSP. FLAP is not necessary for 5LO to bind to membranes: what would a complex formed by a nanodisc and a 5LO look like in its absence? In other words, the structure of 5LO colocalized on the membrane next to FLAP could be different from its structure in a tight complex with FLAP. Moreover, if for example two 5LO would be available per one ND what kind of complexes could form? Would two 5LO bind on the same side of the nanodisc or on opposite sides? Could more than one, two or more 5LO bind per disc? Finally, as one of several mechanisms that could inactivate 5LO (at least *in vitro*) is dimerisation, could the dimer bind on the ND? This would create a complex with the same molecular weight as for two monomer 5LO bound to one ND and complicate structural studies of the 5LO-ND complex (as well as a FLAP-5LO-ND complex later).

### Biophysical and biochemical analysis of monomeric 5LO interactions with ND

In a recent cell based assay an increase in activity was shown concomitant with the 5LO translocation to the membrane [37]. A proximity ligation assay showed 5LO to colocalise with FLAP during the translocation and product formation within a distance less than 40 nm. On a nanodisc the distance would necessarily be less than 40 nm as the available size range is less than 20 nm diameter [21]. Compared to a liposome, the nanodisc provides a piece of soluble membrane with 1) a bilayer accessible from both sides, 2) a small, predefined surface area and 3) a small and homogenous size necessary for methods like native gel electrophoresis and TEM.

A nanodisc with 10.5 nm bilayer diameter was chosen for structural studies of complex formation: it would provide the smallest area that would allow just one FLAP (about 4 nm diameter) and one 5LO (about 4.5 x 9 nm) to bind simultaneously. It may seem tight but on the other hand, minimum extra size would be added compared to the complex of interest. However, 5LO binding on the nuclear membranes depends on the presence of Ca^2+^ not FLAP [38]. Hence, the structure as well as function of the 1:1 complex of 5LO and the ND is interesting in its own right.

Biological molecules containing mostly light elements like carbon, nitrogen and oxygen does not scatter electrons strongly wich results in low contrast when viewed by TEM [34]. Small (<250 kDa) to very small (<100 kDa) particles can be hard to find, if at all, under low contrast conditions, e.g. in vitreous ice made for cryoEM imaging. Hence, staining with strongly scattering heavy metal salts may be the only option to characterize very small biomolecules by TEM. Furthermore, characterization by negative stain TEM of a protein complex selected as a narrow fraction from a size exclusion column and that appears as a single band on a gel may show a heterogenous result. Differences in conformations of the complex constituents or in the complex assembly may be reasons. Characterisation of such details are essential for further investigations of vitrified samples at cryogenic temperatures necessary to reach high resolution structural models. The 5LO monomer and dimer are relatively small molecules whereas the complex of 5LO on ND is large enough for cryoEM.

We used a negative stain (PTA) that has a specific artefactual effect to “stack” phospholipid bilayers [35]. Bearing in mind that all incubations to produce complexes are made on the samples before the negative stain is applied, the stacking by PTA can only be induced in samples where the nanodisc surfaces are free and no large particle is bound (Figure C vs Figures E and F in S1 File).

In several PTA stained samples, it is clear that the nanodiscs were free to bind each other in stacks i.e. Fig 4A–4C and Fig 6A and 6B). Monomer 5LO cannot bind in the absence of calcium ions (Fig 4C) and dimer 5LO cannot bind even with calcium (Fig 6A). The lack of the stacking phenomenon through PTA staining provides an indirect visualization of the protein binding on the nanodisc surface.

In the presence of Ca^2+^, either 1:1 or 2:1 complexes were formed and remained stable when negatively stained, showing well separated single particles by electron microscopy (Fig 4D–4G). For the 1:1 complexes (Fig 4D and 4E), no more than bipartite complexes were observed whereas for the 2:1 complexes (Fig 4F and 4G) tripartite objects can be found. These could represent side views of either one ND with one 5LO embedded (Fig 4D) or one 5LO embedded on each surface of its nanodisc (Fig 4E, see also Figure E and Figure F in S1 File).

This would conform to a model proposed by Pande et al [24]. Based on polarized ATR-FTIR spectroscopy of 5LO on POPC membranes in the presence of Ca^2+^ [23,24] it was suggested that the N-terminal β-sandwich axis of 5LO would form a 45° angle to the membrane and that residues both in the β-sandwich and in the catalytic domain contribute to binding [24]. Furthermore, the knowledge of residues important in membrane binding allowed building a model of 5LO embedded in the membrane [24]. The overall shape of lipoxygenases can be viewed as a cylinder, and in the Pande model 5LO is embedded into the membrane along the cylindrical long axis [24]. These authors showed that although the calculated maximum cross-sectional area of 5LO is about 3500 Å^2^, the effective area per 5LO binding site at the membrane is about 9600±1250 Å^2^. The nanodiscs used for our study have a somewhat smaller bilayer area of about 8600 Å^2^ calculated from the innerdiameter of about 10.5 nm. On the other hand, in case the area would be insufficient, uncomplexed 5LO should be present even when there is an excess ND per 5LO (see the titration Fig 3, lanes 3–6). This is not the case and the ND surface area seems to be sufficient to bind one 5LO.

On the other hand, a large number of the nanodiscs bind only one 5LO even in the presence of an excess 5LO. Possibly, the choice of POPC as the only lipid may not be optimal or even that monomeric 5LO may have a non-binding conformation. Perhaps more likely: the residues in the N-terminal C2-domain probe deeply into the membrane [23,24,39] and could affect the bilayer as was seen for the IVA type cPLA2a C2-domain [40] and for monotopic membrane proteins investigated by molecular dynamics [41,42]. In the worst case, the membrane distorsion could prevent the second 5LO to bind on the opposite ND side.

In fact, one could speculate that even if Ca^2+^ mediates the first level of binding on the phospholipid bilayer, the burial of the loops containing tryptophans 13, 75 and 102 (located on the in the β-sandwich) would be a second level of binding and this would distort the membrane. Or, as a third level of binding, the membrane distorsion is induced only for those ND where also the C-terminal 5LO domain embeds in the ND lipid bilayer. In other words, the burial of the loops could be reinforced further by the C-terminal embedding. This reasoning could partly explain the 340 kDa band still remaining after a Ca^2+^ free gel run (Fig 1A, Lane 3). Binding of 5LO to nanodiscs with larger bilayer area [21] and different lipid compositions is a subject for extended studies.

Previous studies have shown that 5LO enzyme activity is upregulated by phospholipids, usually in the form of liposomes, in the presence of Ca^2+^ [11,43,44]. We needed to verify that the nanodiscs would behave as pure liposomes and not be affected by its small size or the MSP protein or some other aspect. When we compared the effects of nanodiscs and liposomes on 5LO activity in presence of Ca^2+^ and there was no statistically significant difference between the two bilayer mimetics, both regarding initial rate and final amount of products (Table 1).

One statistically significant difference for the two bilayer mimetics was that (in the absence of Ca^2+^) the activity of 5LO with liposomes present in the solution was lower than for ND. If there is no calcium present 5LO remains unbound to membranes and depends on the amount on AA free in solution. Possibly, AA would partition also into the liposome interior solution and thereby become less available for 5LO in the solution.

Would the used amounts of Mg^2+^ present in the activity measurements be activating? At the low concentration of both types of membranes, (ND or PC; 10 μg/ml) and the AA at 20 μM, the low activities we observe (in the absence of Ca^2+)^ corresponds very well with earlier reports [26,45]. An addition of Ca^2+^ in the presence of Mg^2+^ gave a 3.3-fold increase in activity [45]. In the present study, Ca^2+^ was added in the presence of Mg^2+^ and strongly increased the activity of 5LO, emphasizing the high affinity of 5LO for Ca^2+^ compared to Mg^2+^.

Thus, binding of 5LO to nanodiscs is a useful model for the association of this lipoxygenase to natural membranes. Recently, nanodiscs were used to confirm Ca^2+^ dependent membrane binding of 15-lipoxygenase-2 [46]. Another reason for the use of nanodiscs is that possibly the confined area of the nanodisc, even more when the FLAP is located within the bilayer, could be the first *in vitro* system where the activity of a complex between 5LO and FLAP could be studied. FLAP reconstituted into liposomes could induce changes in activity or product profiles but no such experiments have been reported. FLAP in detergent does not seem to interact with 5LO (not shown).

### Analysis of Dimeric 5LO

In measurements where neither the preparation of 5LO or the gels were kept fully reduced it is clear that 1) dimer forms but 2) the active form of 5LO is the monomer (Figs 1 and 2). Monomers and dimers could be separated by SEC for later use. In the initial stage of this study, 2-mercaptoethanol was used in the 5LO preparations, however dimerization was abundant. TCEP has a higher reducing power and specifically reduces only the aminoacids on the surface of proteins [47]. When TCEP was used instead of 2-mercaptoethanol, the amount of dimer formed was considerably reduced (data not shown) and the stability of 5LO was also increased. The presence of glutathione also prevented dimerization of 5LO **[13]**.

In the BN-PAGE analyses (Figs 1A and 5) the protein band appearing at MW 160 kDa is compatible with a 5LO dimer. Investigations of lipoxygenases in dimeric form or even in higher oligomeric states under various conditions are available [13,48,49,50,51] and direct investigation of 5LO dimerisation (156 kDa) was first reported in 2011 both *in vivo* and *in vitro* after induction of dimerization **[13]**. The dimer of 5LO is assembled by disulfide bridges formed by cysteines, C159, C300, C416 and C418 and the proposed dimer model has a head to tail orientation **[13]**. Although, this orientation cannot be verified by our low resolution negative stain data (Fig 7) some class-averages look as if two equally long objects are attached with their long dimensions in parallel in support of the model proposed in [13] (see also Figure D2 in S1 File).

We determined the possible interaction of dimeric 5LO with nanodiscs. The results showed no Ca^2+^ mediated binding of dimeric 5LO to ND, either by native gels or TEM (Figs 5 and 6). It can be argued that binding of the dimer on a ND could have other dependencies than calcium ions and be of lower affinity (cf. Figs 1 and 5). Hence this complex would not be stable under the conditions of the native gel experiment. The extensive stacking present in samples where the dimer had been incubated with nanodiscs, both with and without calcium, contradicts formation of stable complexes however (Fig 6). Another possibility for complex formation would have been if the phospholipid bilayer somehow would affect the dimer to separate into monomers that in turn would have been able to bind on the ND. In this case, we should have seen bands corresponding to monomeric 5LO and 430 kDa in Fig 5 and in Fig 6A less stacking and 5LO-ND complexes compared to Fig 6B. This is not the case.

Apart from not being able to bind to nanodiscs, we also noticed that the dimeric 5LO did not show any enzymatic activity (Table A in S1 File), in accordance with earlier work [13]. In the proposed head to tail 5LO dimer model, dimerization causes Trp75 to become sterically hindered for membrane interaction [24]. Furthermore, in the dimer the catalytic sites may face each other in a way that renders them inaccessible to substrate and membrane. If 5LO could be considered to have the shape of a cylinder, the class averages (Fig 4E) support an interaction of monomeric 5LO via the long dimension (that would be embedded on the membrane), rather than via one of the ends of the cylinder.

It was reported that 11*R*-lipoxygenase was present as a dimer in calcium-free buffer and in the presence of 10 mM CaCl_2_ large aggregates were formed [51]. Could the incubation with calcium be the reason for 5LO dimer-formation from monomer or dissociation of dimer to monomer? For the 5LO monomer, calcium did not promote increased dimerisation (Fig 1A). Also, BN-PAGE analysis indicated no change of the dimeric state, by Ca^2+^ (Fig 5).

## Conclusion

In summary, we can replace liposomes with nanodiscs, they function equally well as membrane mimetics. The activities and products formed are very similar. We have obtained negative stain images of complexes from different angles with either one or two 5LO bound to one nanodisc. The native page and negative stain results also show that the preparation of a pure specimen for cryoEM is straightforward; incubation (with Ca^2+^) of a monomeric 5LO with nanodiscs at a ratio 1:1 will result in a majority of 1:1 complexes. Moreover, for all complexes of 5LO-ND it is the monomeric 5LO that binds. In case there would be a small fraction of dimers present in an otherwise monomeric preparation of 5LO, these would remain unbound. Possibly speculative but in some class-averages representing the two 5LO per disc it seemed that the two 5LO bound on the same side of the nanodisc. With this in mind, the chosen nanodisc size should be able to house the complex between FLAP and 5LO as calculated and observed preliminary (not shown). For Single Particle Reconstruction (SPR), the 1:1 complex of 5LO-ND (340 kDa) approaches the medium size range. This means the complex should be detectable in vitrified ice and data could be collected by cryoEM to reconstruct a structure of the complex, possibly with and without the presence of FLAP.

## Supporting Information

S1 File (PDF)

## References

[1] Haeggstrom JZ, Wetterholm A (2002) Enzymes and receptors in the leukotriene cascade. Cell Mol Life Sci 59: 742–753. 12088275 10.1007/s00018-002-8463-1 PMC11146160

[2] Radmark O, Werz O, Steinhilber D, Samuelsson B (2014) 5-Lipoxygenase, a key enzyme for leukotriene biosynthesis in health and disease. Biochim Biophys Acta. 10.1016/j.bbalip.2014.08.012 25152163

[3] Gilbert NC, Bartlett SG, Waight MT, Neau DB, Boeglin WE, et al (2011) The structure of human 5-lipoxygenase. Science 331: 217–219. 10.1126/science.1197203 21233389 PMC3245680

[4] Jakschik BA, Sun FF, Lee L, Steinhoff MM (1980) Calcium stimulation of a novel lipoxygenase. Biochem Biophys Res Commun 95: 103–110. 6251797 10.1016/0006-291x(80)90710-x

[5] Radmark O, Samuelsson B (2010) Regulation of the activity of 5-lipoxygenase, a key enzyme in leukotriene biosynthesis. Biochem Biophys Res Commun 396: 105–110. 10.1016/j.bbrc.2010.02.173 20494120

[6] Rouzer CA, Samuelsson B (1987) 5-Lipoxygenase from human leukocytes associates with membrane in the presence of calcium. Adv Prostaglandin Thromboxane Leukot Res 17A: 60–63. 2959118

[7] Zhang YY, Hammarberg T, Radmark O, Samuelsson B, Ng CF, et al (2000) Analysis of a nucleotide-binding site of 5-lipoxygenase by affinity labelling: binding characteristics and amino acid sequences. Biochem J 351 Pt 3: 697–707. 11042125 PMC1221410

[8] Miller DK, Gillard JW, Vickers PJ, Sadowski S, Leveille C, et al (1990) Identification and isolation of a membrane protein necessary for leukotriene production. Nature 343: 278–281. 2300172 10.1038/343278a0

[9] Reid GK, Kargman S, Vickers PJ, Mancini JA, Leveille C, et al (1990) Correlation between expression of 5-lipoxygenase-activating protein, 5-lipoxygenase, and cellular leukotriene synthesis. J Biol Chem 265: 19818–19823. 2174053

[10] Basavarajappa D, Wan M, Lukic A, Steinhilber D, Samuelsson B, et al (2014) Roles of coactosin-like protein (CLP) and 5-lipoxygenase-activating protein (FLAP) in cellular leukotriene biosynthesis. Proc Natl Acad Sci U S A 111: 11371–11376. 10.1073/pnas.1410983111 25034252 PMC4128154

[11] Rakonjac M, Fischer L, Provost P, Werz O, Steinhilber D, et al (2006) Coactosin-like protein supports 5-lipoxygenase enzyme activity and up-regulates leukotriene A4 production. Proc Natl Acad Sci U S A 103: 13150–13155. 16924104 10.1073/pnas.0605150103 PMC1559768

[12] Lu W, Zhao X, Xu Z, Dong N, Zou S, et al (2013) Development of a new colorimetric assay for lipoxygenase activity. Anal Biochem 441: 162–168. 10.1016/j.ab.2013.06.007 23811155

[13] Hafner AK, Cernescu M, Hofmann B, Ermisch M, Hornig M, et al (2011) Dimerization of human 5-lipoxygenase. Biol Chem 392: 1097–1111. 10.1515/BC.2011.200 22050225

[14] Skorey KI, Gresser MJ (1998) Calcium is not required for 5-lipoxygenase activity at high phosphatidyl choline vesicle concentrations. Biochemistry 37: 8027–8034. 9609696 10.1021/bi980371g

[15] Noguchi M, Miyano M, Kuhara S, Matsumoto T, Noma M (1994) Interfacial kinetic reaction of human 5-lipoxygenase. Eur J Biochem 222: 285–292. 8020467 10.1111/j.1432-1033.1994.tb18867.x

[16] Wong A, Hwang SM, Cook MN, Hogaboom GK, Crooke ST (1988) Interactions of 5-lipoxygenase with membranes: studies on the association of soluble enzyme with membranes and alterations in enzyme activity. Biochemistry 27: 6763–6769. 3143404 10.1021/bi00418a018

[17] Jiang QX, Chester DW, Sigworth FJ (2001) Spherical reconstruction: a method for structure determination of membrane proteins from cryo-EM images. J Struct Biol 133: 119–131. 11472084 10.1006/jsbi.2001.4376

[18] Liu Y, Sigworth FJ (2014) Automatic cryo-EM particle selection for membrane proteins in spherical liposomes. J Struct Biol 185: 295–302. 10.1016/j.jsb.2014.01.004 24468290 PMC3978669

[19] Wang L, Sigworth FJ (2009) Structure of the BK potassium channel in a lipid membrane from electron cryomicroscopy. Nature 461: 292–295. 10.1038/nature08291 19718020 PMC2797367

[20] Denisov IG, Grinkova YV, Lazarides AA, Sligar SG (2004) Directed self-assembly of monodisperse phospholipid bilayer Nanodiscs with controlled size. J Am Chem Soc 126: 3477–3487. 15025475 10.1021/ja0393574

[21] Grinkova YV, Denisov IG, Sligar SG (2010) Engineering extended membrane scaffold proteins for self-assembly of soluble nanoscale lipid bilayers. Protein Eng Des Sel 23: 843–848. 10.1093/protein/gzq060 20817758 PMC2953958

[22] Bayburt TH, Sligar SG (2010) Membrane protein assembly into Nanodiscs. FEBS Lett 584: 1721–1727. 10.1016/j.febslet.2009.10.024 19836392 PMC4758813

[23] Pande AH, Moe D, Nemec KN, Qin S, Tan S, et al (2004) Modulation of human 5-lipoxygenase activity by membrane lipids. Biochemistry 43: 14653–14666. 15544336 10.1021/bi048775y

[24] Pande AH, Qin S, Tatulian SA (2005) Membrane Fluidity Is a Key Modulator of Membrane Binding, Insertion, and Activity of 5-Lipoxygenase. Biophys J 88: 4084–4094. 15778441 10.1529/biophysj.104.056788 PMC1305639

[25] Zhang YY, Hamberg M, Radmark O, Samuelsson B (1994) Stabilization of purified human 5-lipoxygenase with glutathione peroxidase and superoxide dismutase. Anal Biochem 220: 28–35. 7978252 10.1006/abio.1994.1294

[26] Percival MD, Denis D, Riendeau D, Gresser MJ (1992) Investigation of the mechanism of non-turnover-dependent inactivation of purified human 5-lipoxygenase. Inactivation by H2O2 and inhibition by metal ions. Eur J Biochem 210: 109–117. 1446663 10.1111/j.1432-1033.1992.tb17397.x

[27] Bradford MM (1976) A rapid and sensitive method for the quantitation of microgram quantities of protein utilizing the principle of protein-dye binding. Anal Biochem 72: 248–254. 942051 10.1016/0003-2697(76)90527-3

[28] Denisov IG, Baas BJ, Grinkova YV, Sligar SG (2007) Cooperativity in cytochrome P450 3A4: linkages in substrate binding, spin state, uncoupling, and product formation. J Biol Chem 282: 7066–7076. 17213193 10.1074/jbc.M609589200

[29] Tang G, Peng L, Baldwin PR, Mann DS, Jiang W, et al (2007) EMAN2: an extensible image processing suite for electron microscopy. J Struct Biol 157: 38–46. 16859925 10.1016/j.jsb.2006.05.009

[30] Radermacher M, Ruiz T (2006) Three-dimensional reconstruction of single particles in electron microscopy image processing. Methods Mol Biol 319: 427–461. 16719367 10.1007/978-1-59259-993-6_20

[31] Brooks SP, Storey KB (1992) Bound and determined: a computer program for making buffers of defined ion concentrations. Anal Biochem 201: 119–126. 1621949 10.1016/0003-2697(92)90183-8

[32] Ritchie TK, Grinkova YV, Bayburt TH, Denisov IG, Zolnerciks JK, et al (2009) Chapter 11 Reconstitution of Membrane Proteins in Phospholipid Bilayer Nanodiscs In: Nejat D, editor. Methods in Enzymology: Academic Press pp. 211–231. 10.1016/S0076-6879(09)64011-8 PMC4196316 19903557

[33] Hammarberg T, Radmark O (1999) 5-lipoxygenase binds calcium. Biochemistry 38: 4441–4447. 10194365 10.1021/bi9824700

[34] Ohi M, Li Y, Cheng Y, Walz T (2004) Negative Staining and Image Classification—Powerful Tools in Modern Electron Microscopy. Biol Proced Online 6: 23–34. 15103397 10.1251/bpo70 PMC389902

[35] Zhang L, Song J, Cavigiolio G, Ishida BY, Zhang S, et al (2011) Morphology and structure of lipoproteins revealed by an optimized negative-staining protocol of electron microscopy. J Lipid Res 52: 175–184. 10.1194/jlr.D010959 20978167 PMC2999936

[36] Kucerka N, Nieh MP, Katsaras J (2011) Fluid phase lipid areas and bilayer thicknesses of commonly used phosphatidylcholines as a function of temperature. Biochim Biophys Acta 1808: 2761–2771. 10.1016/j.bbamem.2011.07.022 21819968

[37] Gerstmeier J, Weinigel C, Rummler S, Radmark O, Werz O, et al (2016) Time-resolved in situ assembly of the leukotriene-synthetic 5-lipoxygenase/5-lipoxygenase-activating protein complex in blood leukocytes. FASEB J 30: 276–285. 10.1096/fj.15-278010 26396238

[38] Chen XS, Funk CD (2001) The N-terminal "beta-barrel" domain of 5-lipoxygenase is essential for nuclear membrane translocation. J Biol Chem 276: 811–818. 11042185 10.1074/jbc.M008203200

[39] Kulkarni S, Das S, Funk CD, Murray D, Cho W (2002) Molecular basis of the specific subcellular localization of the C2-like domain of 5-lipoxygenase. J Biol Chem 277: 13167–13174. 11796736 10.1074/jbc.M112393200

[40] Ward KE, Ropa JP, Adu-Gyamfi E, Stahelin RV (2012) C2 domain membrane penetration by group IVA cytosolic phospholipase A(2) induces membrane curvature changes. J Lipid Res 53: 2656–2666. 10.1194/jlr.M030718 22991194 PMC3494261

[41] Jaud S, Tobias DJ, Falke JJ, White SH (2007) Self-induced docking site of a deeply embedded peripheral membrane protein. Biophys J 92: 517–524. 17071664 10.1529/biophysj.106.090704 PMC1751407

[42] Balali-Mood K, Bond PJ, Sansom MS (2009) Interaction of monotopic membrane enzymes with a lipid bilayer: a coarse-grained MD simulation study. Biochemistry 48: 2135–2145. 10.1021/bi8017398 19161285

[43] Puustinen T, Scheffer MM, Samuelsson B (1988) Regulation of the human leukocyte 5-lipoxygenase: stimulation by micromolar Ca2+ levels and phosphatidylcholine vesicles. Biochim Biophys Acta 960: 261–267. 3382674 10.1016/0005-2760(88)90033-1

[44] Noguchi M, Miyano M, Matsumoto T, Noma M (1994) Human 5-lipoxygenase associates with phosphatidylcholine liposomes and modulates LTA4 synthetase activity. Biochim Biophys Acta 1215: 300–306. 7811715 10.1016/0005-2760(94)90057-4

[45] Reddy KV, Hammarberg T, Radmark O (2000) Mg2+ activates 5-lipoxygenase in vitro: dependency on concentrations of phosphatidylcholine and arachidonic acid. Biochemistry 39: 1840–1848. 10677235 10.1021/bi9919246

[46] Kobe MJ, Neau DB, Mitchell CE, Bartlett SG, Newcomer ME (2014) The structure of human 15-lipoxygenase-2 with a substrate mimic. J Biol Chem 289: 8562–8569. 10.1074/jbc.M113.543777 24497644 PMC3961679

[47] Antia NJ, Chorney V (1967) Nature of the aldolase acitivity in a unicellular red alga. Nature 214: 1028–1029. 6055399 10.1038/2141028a0

[48] Aleem AM, Jankun J, Dignam JD, Walther M, Kuhn H, et al (2008) Human platelet 12-lipoxygenase, new findings about its activity, membrane binding and low-resolution structure. J Mol Biol 376: 193–209. 18155727 10.1016/j.jmb.2007.11.086

[49] Aleem AM, Wells L, Jankun J, Walther M, Kuhn H, et al (2009) Human platelet 12-lipoxygenase: naturally occurring Q261/R261 variants and N544L mutant show altered activity but unaffected substrate binding and membrane association behavior. Int J Mol Med 24: 759–764. 19885615 10.3892/ijmm_00000289

[50] Shang W, Ivanov I, Svergun DI, Borbulevych OY, Aleem AM, et al (2011) Probing dimerization and structural flexibility of mammalian lipoxygenases by small-angle X-ray scattering. J Mol Biol 409: 654–668. 10.1016/j.jmb.2011.04.035 21530540

[51] Eek P, Jarving R, Jarving I, Gilbert NC, Newcomer ME, et al (2012) Structure of a calcium-dependent 11R-lipoxygenase suggests a mechanism for Ca2+ regulation. J Biol Chem 287: 22377–22386. 10.1074/jbc.M112.343285 22573333 PMC3381197

