# Supplementary material for: Structural and Functional Analysis of Calcium Ion Mediated Binding of 5-Lipoxygenase to Nanodiscs
Source: PLoS One. 2016 Mar 24;11(3):e0152116. doi: 10.1371/journal.pone.0152116 (PMC4806843; doi:10.1371/journal.pone.0152116)
Supplement: S1 File — (PDF) [file pone.0152116.s001.pdf]

S1 File: (**A-F**) Schematic Figures of objects discussed in the main text. (**G**) Result from UF staining of a two 5LO per ND incubated with Ca^{2+}. (**H**) Table A showing the results from activity measurements when the dimer 5LO was present instead of the monomer 5LO.

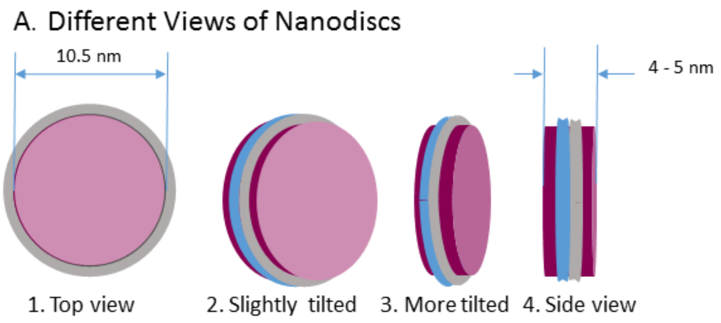

A. Various possible orientations of nanodiscs (ND) made with MSP1E3D1-POPC. The size and shape of ND viewed from top (A1). In the side view (A4) an approximate bilayer thickness is indicated. One of the two MSP protein-chains is coloured grey and the other with blue. The phospholipid bilayer is represented by the thin cylinder (in dark plum colour).

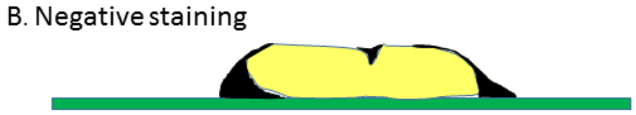

B. The compounds used for negative staining in electron microscopy are not supposed to interact chemically with the target. The negative staining arises when the small grains of the stain cover some crevices and areas around the specimen, so that the image resembles a photographic negative. Uranyl Formate has the smallest grain size of about 0.3 nm. Here Fig B shows a cut through an object (yellow) on carbon film (green) covered with stain (black).

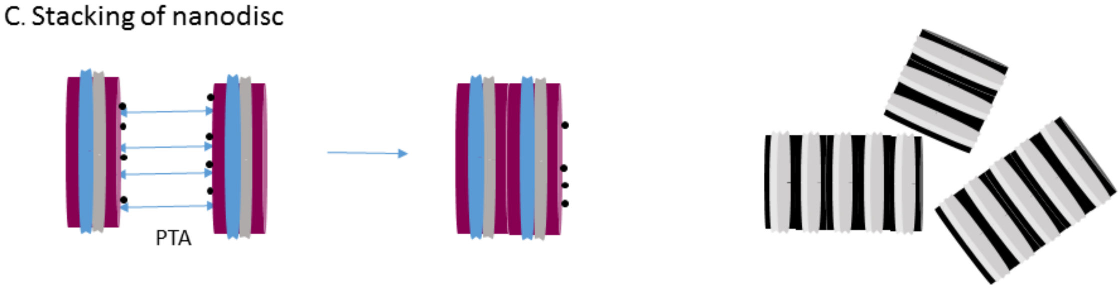

C. Phosphotungstic acid (PTA) can have a chemical interaction with phospholipids (1), which is observed also in the case of nanodiscs since they contain phospholipids. As indicated by blue arrows, PTA molecules interact with the phospholipid bilayer (Left) so that the two lipid surfaces stack up onto each other (Middle. Black dots; a few PTA grains). This can lead to long ”stacks” also called ”rouleaux” (Right, now in black and white as present in main text Fig 4 A-C and Fig. 6 A,B) (1). Uranyl Formate does not interact with the ND bilayers and staining would show top views as in (A1).

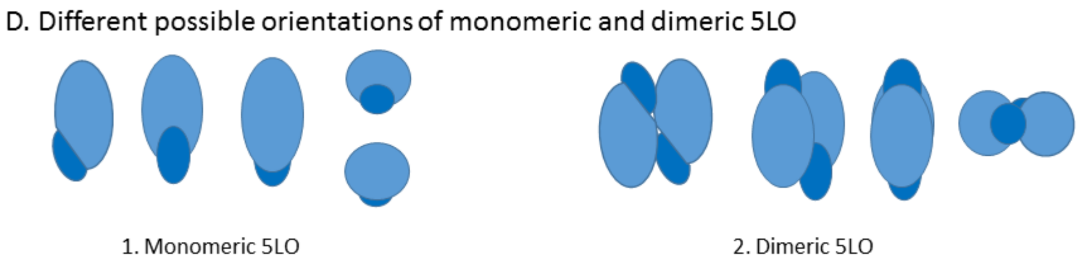

D. Different views of monomeric 5LO (D1) and dimeric 5LO (D2). The N-terminal β-sandwich (darker blue) contains the tryptophans that binds at an angle of 45° to the membrane (2). The C-terminal α-helical larger part of 5LO (pale blue) contains the catalytic site that faces the membrane (3). The dimer schematic (D2) follows the proposed assembly by Häfner et al (4) for 5LO under oxidizing conditions. Cysteines located on the same face of 5LO as the catalytic site form disulfide bridges with cysteines in the opposite 5LO (4). Hereby, the catalytic site is protected from access, hence the enzyme cannot be active (see below, Table A). Furthermore, also the tryptophans would be prevented from access to the membrane, hence no membrane binding can occur (Fig. 6B) even in the presence of calcium (Fig. 6A).

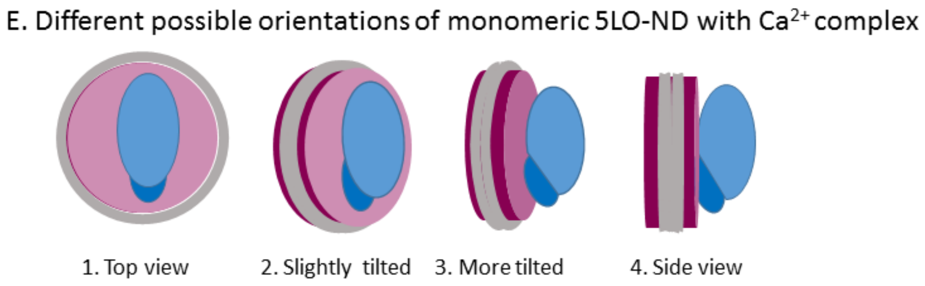

E. One 5LO bound on a nanodisc (E1) gradually tilted towards a side view (E4) of the complex. This scheme is an attempt to illustrate some of the class-averages obtained for the 1:1 complex formed with calcium (Fig. 4E, main text). The lower right corner view of the class averages could be a side view of the complex as depicted in schematic view E4.

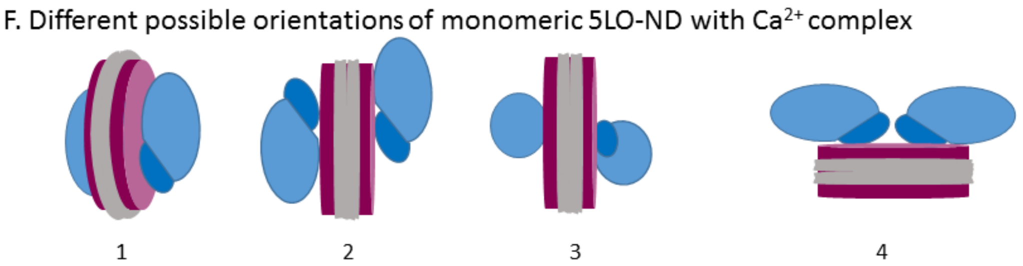

F. For two 5LO binding to one ND many possibilities could be imagined. Here a few ideas. Both a top view and slightly tilted ND with two 5LO would look similar to those carrying only one 5LO (E1 and E2). The two middle schematics (C2, C3) show two nanodisc side views. The orientation of 5LO may vary relative to each other, here depicted as oppositely oriented side views (C2) or as views down their long axis (C3). To the right (C4) what would occur if two 5LO would bind on the same side of the ND: they would not be able to embed the C-terminal part in the membrane, only the N-terminal β-sandwich. On the other hand, it is tryptophan in the β-sandwich that mediate the Ca^{2+} dependent membrane binding (5).

G. More than two 5LO per nanodisc in the presence of calcium stained with Uranyl Formate

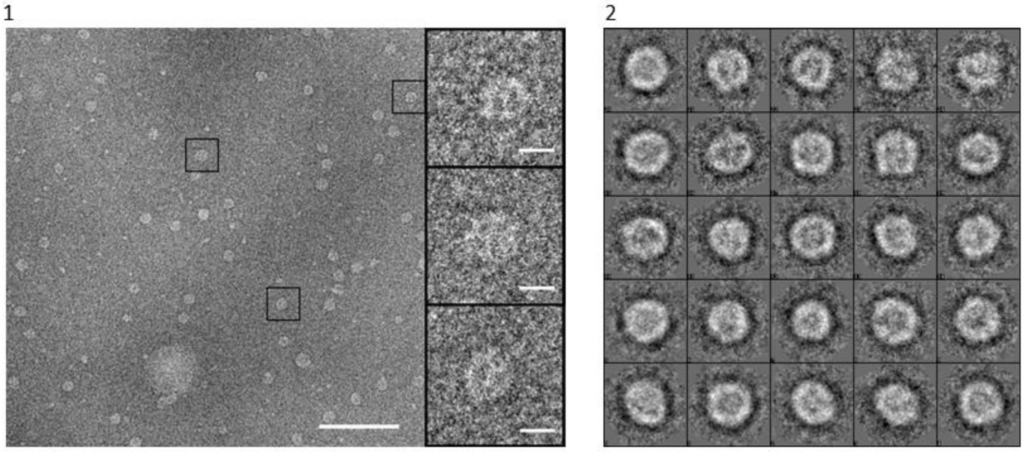

G. Calcium induced binding of two 5LO per one nanodisc.

ND and 5LO were incubated at a 1:2 (ND: 5LO) molar ratio and stained by uranyl formate (UF) instead of PTA (cf. main text Fig. 4D, E). (G1) shows both raw image and three boxed particles, magnified to the right, all with a size larger than 10 nm. (G2) 25 Class-averages were made from 553 particles from images like and including G1. With the numbering 0 lowest left corner and 24 in the top right corner, 18 (and 22) could represent a "tripartite" side view of a nanodisc with one 5LO on each bilayer surface (see F2 above). Possibly 0 and 13 are slightly tilted views (e.g. E2, E3 but also F1) but most classes look like top views for which it is hard to say whether a 5LO is located on the surface (E1) or if it is a ND not bound to 5LO (A1). Possibly nanodiscs in a uranyl stain has a preference in orientation, so that side views of the 1:1 complex ("bipartite") are not present. The UF stain did not really show better resolution, however, it indicates complexes formed are present also in another stain than the PTA.

Table A. Dimeric 5LO activity assay

Assay Components	5HETE/5 HPETE µmol/mg	LT µmol/ mg
**Dimer 5LO + AA**		
With Ca^{2+}	n.d	n.d
Without Ca^{2+}	n.d	n.d
**Dimer 5LO + AA + ND**		
With Ca^{2+}	n.d	n.d
Without Ca^{2+}	n.d	n.d
**Dimer 5LO + AA + PC**		
With Ca^{2+}	n.d	n.d
Without Ca^{2+}	n.d	n.d

Table A. Activity of Dimeric 5LO measured by LC-MS/MS. Total product (5HPETE/5HETE or LTA4) formed in 10 min incubations with different activating factors (conditions as for main text Table 1). N.d - Not detected. Data are mean±SEM (n=3).

References

1. Zhang L, Song J, Cavigiolio G, Ishida BY, Zhang S, Kane JP, et al. Morphology and structure of lipoproteins revealed by an optimized negative-staining protocol of electron microscopy. J Lipid Res. 2011 Jan;52(1):175-84. PubMed PMID: 20978167. Pubmed Central PMCID: 2999936. Epub 2010/10/28. eng.
2. Pande AH, Qin S, Tatulian SA. Membrane fluidity is a key modulator of membrane binding, insertion, and activity of 5-lipoxygenase. Biophys J. 2005 Jun;88(6):4084-94. PubMed PMID: 15778441. Pubmed Central PMCID: 1305639. Epub 2005/03/22. eng.
3. Pande AH, Moe D, Nemec KN, Qin S, Tan S, Tatulian SA. Modulation of human 5-lipoxygenase activity by membrane lipids. Biochemistry. 2004 Nov 23;43(46):14653-66. PubMed PMID: 15544336. Epub 2004/11/17. eng.
4. Hafner AK, Cernescu M, Hofmann B, Ermisch M, Hornig M, Metzner J, et al. Dimerization of human 5-lipoxygenase. Biol Chem. 2011 Dec;392(12):1097-111. PubMed PMID: 22050225. Epub 2011/11/05. eng.
5. Wong A, Hwang SM, Cook MN, Hogaboom GK, Crooke ST. Interactions of 5-lipoxygenase with membranes: studies on the association of soluble enzyme with membranes and alterations in enzyme activity. Biochemistry. 1988 Sep 6;27(18):6763-9. PubMed PMID: 3143404. Epub 1988/09/06. eng.
